# Noasaurids are a component of the Australian ‘mid’-Cretaceous theropod fauna

**DOI:** 10.1038/s41598-020-57667-7

**Published:** 2020-01-29

**Authors:** Sienna A. Birch, Elizabeth T. Smith, Phil R. Bell

**Affiliations:** 1 School of environmental and Rural Science, University of New England, Armidale, NSW Australia; 2 Australian Opal Centre, Lightning Ridge, NSW Australia

**Keywords:** Palaeontology, Phylogenetics

## Abstract

The diversity of Australia’s theropod fauna from the ‘mid’-Cretaceous (Albian–Cenomanian) is distinctly biased towards the medium-sized megaraptorids, despite the preponderance of abelisauroids in the younger but latitudinally equivalent Patagonian theropod fauna. Here, we present new evidence for the presence of ceratosaurian, and specifically abelisauroid, theropods from the Cenomanian Griman Creek Formation of Lightning Ridge, New South Wales. A partial cervical vertebra is described that bears a mediolaterally concave ventral surface of the centrum delimited by sharp ventrolateral ridges that contact the parapophyses. Among theropods, this feature has been reported only in a cervical vertebra attributed to the noasaurid *Noasaurus*. We also reappraise evidence recently cited against the ceratosaurian interpretation of a recently described astragalocalcaneum from the upper Barremian–lower Aptian San Remo Member of the upper Strzelecki Group in Victoria. Inclusion of the Lightning Ridge cervical vertebra and Victorian astragalocalcaneum into a revised phylogenetic analysis focused on elucidating ceratosaurian affinities reveals support for placement of both specimens within Noasauridae, which among other characters is diagnosed by the presence of a medial eminence on the ascending process of the astragalus. The Lightning Ridge and Victorian specimens simultaneously represent the first noasaurids reported from Australia and the astragalocalcaneum is considered the earliest known example of a noasaurid in the world to date. The recognition of Australian noasaurids further indicates a more widespread Gondwanan distribution of the clade outside of South America, Madagascar and India consistent with the timing of the fragmentation of the supercontinent.

## Introduction

The composition of Australia’s theropod fauna is poorly understood in comparison to those of contemporaneous assemblages around the world, due primarily to the isolated and fragmentary mode of preservation in fossiliferous deposits. To date, the majority of documented theropod remains from Australia are from the ‘mid’-Cretaceous (Albian–Cenomanian) and pertain predominantly to megaraptorids^1–7^, an exclusively Gondwanan clade of theropods initially interpreted as a member of Allosauroidea^2^. However, recent hypotheses have suggested alternative positions for megaraptorids within Tyrannosauroidea^8–11^ or close to the base of Coelurosauria^12,13^. Despite the preponderance of megaraptorids in ‘mid’-Cretaceous Australia, a diverse high palaeo-latitude (approximately 60 degrees south) theropod fauna has been hypothesised within the upper Barremian–lower Albian deposits on the south coast of Victoria, including megaraptorans^3,5,14^, ceratosaurs^15^, spinosaurids^16^, tyrannosauroids^3,17^, possible unenlagiine dromaeosaurids and indeterminate maniraptoriforms^3^.

While members of Avetheropoda were undoubtedly present during the Cretaceous of Australia, the evidence for Ceratosauria in Australia is presently very limited, despite their abundance in the diverse Patagonian theropod fossil record^8^. The first suggested Australian ceratosaur came not from the better known Cretaceous sites in eastern Australia, but from the Middle Jurassic Colalura Sandstone of Western Australia. *Ozraptor subotaii* was described from a distal tibia characterised by a depressed and subdivided facet for the ascending process of the astragalus^18^. Examination of the tibial fragment failed to identify any convincing similarities with any theropod known at the time, and thus *Ozraptor* was referred to as an indeterminate theropod^17^. Subsequently, the description of abelisauroid remains from the Late Jurassic of Africa included tibiae that also had astragalar articular surfaces similar to that of *Ozraptor*. On this basis, it was suggested that the Australian tibia represented a member of Abelisauroidea^19^. This interpretation was maintained in a reassessment of a theropod distal tibia from the Middle Jurassic of England^20^, which concluded that a depressed and subdivided facet for the astragalar ascending process was a synapomorphy of Abelisauroidea. However, this character was subsequently recognised in theropods outside of Abelisauroidea and therefore could not be considered as an abelisauroid synapomorphy^21^. As a consequence there was no convincing evidence to support abelisauroid affinities for *Ozraptor*. The current consensus is that *Ozraptor* is too incomplete for referral to any theropod clade^21,22^.

There has also been suggestion that *Kakuru kujani*, known from a partial tibia from the Aptian Marree Formation of South Australia^23^ pertains to an abelisauroid based on the presence of a vertical median ridge on the distal tibia^24^. For the reasons stated above, this evidence is insufficient for referral of *Kakuru* to Abelisauroidea; subsequent revisions of this material concluded that *Kakuru* could only be referred to an indeterminate position within either Averostra or Tetanurae^25,26^.

**Figure 1.**
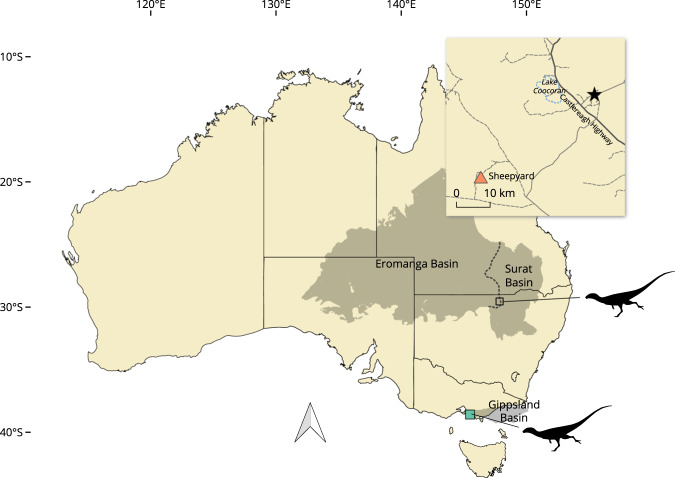
Map of Australia showing the location of Lightning Ridge and the Sheepyard opal field in central north New South Wales (inset) and San Remo in southern Victoria. The Eromanga, Surat and Gippsland basins are represented by the grey areas; the dashed line indicates the boundary between the Eromanga and Surat basins. The inset map, indicated by the square on the main map, shows location of the Sheepyard opal field (orange triangle) where LRF 3050.AR was found in the vicinity of Lightning Ridge (black star). The location of San Remo on the south coast of Victoria, the area in which NMV P221202 was discovered, is indicated by the green square. Australia coastline uses data taken from GEODATA COAST 100 K 2004 provided by Geoscience Australia (http://www.ga.gov.au/metadata-gateway/metadata/record/61395). Basin extents data from Australian Geological Provinces, 2013.01 edition (http://www.ga.gov.au/metadata-gateway/metadata/record/74371/); both released by Geoscience Australia under CC BY 4.0 license (https://creativecommons.org/licenses/by/4.0/). Silhouette by Tasman Dixon and released under a Public Domain Dedication 1.0 license (http://creativecommons.org/publicdomain/zero/1.0/). Roads and geographic map data ^©^ OpenStreetMap contributors (https://www.openstreetmap.org); data made available under the Open Database License (https://www.opendatacommons.org/licenses/odbl).

More recently, a left astragalocalcaneum from the upper Barremian–lower Albian San Remo Member of the upper Strzelecki Group on the south coast of Victoria was described (Museum Victoria, Melbourne, Australia; NMV P221202, Fig. 1) and referred to Ceratosauria, based among other features on the co-ossification of the astragalus and calcaneum, a parallel-sided base of the ascending process of the astragalus, and a fossa at the base of the ascending process that is not associated with a transverse groove^15^. However, it was subsequently suggested that the evidence for referral of NMV P221202 to Ceratosauria was weak, and that it could only be considered as an indeterminate averostran at best^8^.

Here, we present new evidence for the presence of ceratosaurian theropods from the Cenomanian Griman Creek Formation of Lightning Ridge, New South Wales. We also reappraise the evidence against the ceratosaurian interpretation of the specimen NMV P221202^8^ with the objective of clarifying and elucidating its phylogenetic position.

## Taxonomic Framework

There are presently two hypotheses regarding the content of Noasauridae and the phylogeny of non-abelisaurid, non-ceratosaurid ceratosaurians. Abelisauroidea was originally considered to include Abelisauridae and Noasauridae, and all ceratosaurs more closely related to them than to *Ceratosaurus nasicornis*^27^. The earliest phylogenetic analysis of ceratosaurs identified a monophyletic Abelisauroidea following this definition^28^, and which was subsequently expanded to include the African *Elaphrosaurus bambergi*^29^. Subsequent phylogenetic studies expanded the taxonomic scope of Noasauridae to include small-bodied Late Cretaceous taxa from South America^21,30–32^ and the Jurassic and Cretaceous of Africa^33^, to the exclusion of *Elaphrosaurus*. This topology has been widely recovered in more recent analyses^21,34–39^. However, *Elaphrosaurus* has also been resolved within Noasauridae in other analyses^40^, most notably in the analysis accompanying the recent redescription of the holotype^41^. Under this hypothesis, the subclade Noasaurinae was coined to include ceratosaurs more closely related to *Noasaurus leali* than to *Elaphrosaurus*, *Ceratosaurus* and *Allosaurus fragilis*, and Elaphrosaurinae was erected to include ceratosaurs more closely related to *Elaphrosaurus* than to *Noasaurus*, *Abelisaurus comahuensis*, *Ceratosaurus* and *Allosaurus*^41^. The results of a revised phylogenetic analysis for *Limusaurus inextricabilis*^42^ were used to support a recently proposed phylogenetic framework for Ceratosauria^43^ in which Noasaurinae and Elaphrosaurinae were recovered as subclades of Noasauridae. In line with the topology of our phylogenetic tree (see Phylogenetic Analysis), the following descriptions and discussions consider Noasauridae to have the same taxonomic content as Noasaurinae^41^, with members of Elaphrosaurinae representing ceratosaurs basal to Abelisauroidea (i.e., Noasauridae + Abelisauridae).

## Systematic Palaeontology

Theropoda Marsh 1881

Neotheropoda Bakker 1986

Averostra Paul 2002

Ceratosauria Marsh 1884

Noasauridae indet. Bonaparte and Powell 1980^44^

### LRF 3050.AR

#### Locality

LRF (Australian Opal Centre, Lightning Ridge, New South Wales, Australia) 3050.AR was collected from an underground opal mine at the ‘Sheepyard’ opal field, approximately 40 km southwest of Lightning Ridge in central northern New South Wales (Fig. 1). The specimen derives from the Wallangulla Sandstone Member^45^ of the Griman Creek Formation. Radiometric dates for the Wallangulla Sandstone Member at Lightning Ridge indicate a maximum depositional age of 100.2–96.6 Ma^46^. LRF 3050.AR was found within a monodominant bonebed of the iguanodontian *Fostoria dhimbangunmal*^47^. Other faunal components from this accumulation include isolated unionid bivalves (LRF 3051), a testudine caudal vertebra (LRF 3053), a small ornithopod caudal centrum (LRF 3052), and a possible indeterminate theropod ulna (LRF 3054). A complete discussion of the geological setting, sedimentology, age and faunal diversity of the Griman Creek Formation is presented elsewhere^46^.

#### Description

**Figure 2.**
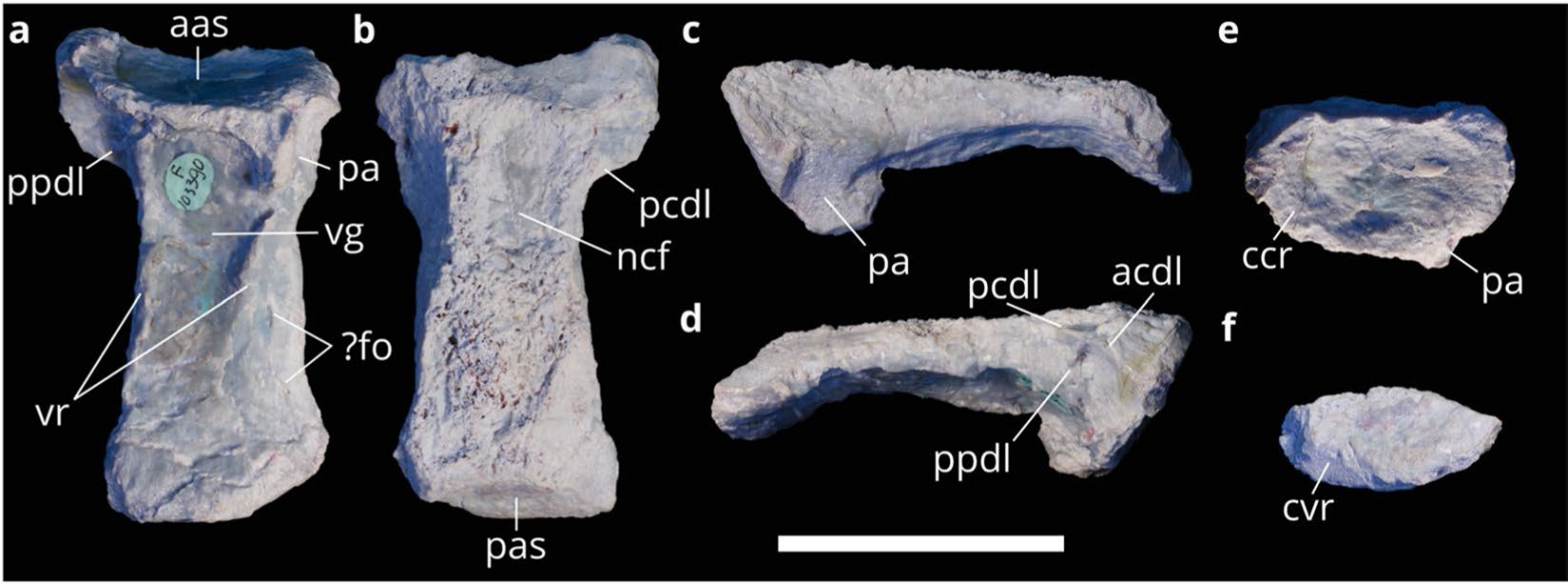
The cervical vertebra LRF 3050.AR in (**a**) ventral; (**b**) dorsal, (**c**) left lateral, (**d**) right lateral, (**e**) anterior and (**f**) posterior views. Abbreviations: aas, anterior articular surface; acdl, anterior centrodiapophyseal lamina; ccr, concave rim; cvr, convex rim; fo, foramina; ncf, floor of the neural canal; pa, parapophysis; pas, posterior articular surface; pcdl, posterior centrodiapophyseal lamina; ppdl, paradiapophyseal lamina, vg, ventral depression; vr, ventrolateral ridge. Scale bar equals 50 mm.

LRF 3050.AR has been taphonomically altered by erosion, breakage and through preparation. The centrum is markedly flattened dorsoventrally through taphonomic compaction, such that much of the left lateral surface is visible in ventral view. In addition, the dorsal portion of the centrum has been sheared off obliquely. Notwithstanding the dorsoventral compression, the centrum is hourglass-shaped in dorsal-ventral view; the narrowest point occurs approximately one-third of the length from the anterior articular surface (Fig. 2a,b). In lateral view, the anterior and posterior articular surfaces are oriented obliquely relative to the long axis of the centrum (approximately 20 degrees from vertical; Fig. 2c,d); however, this appearance is probably a result of the taphonomic compaction and not indicative of their original orientations. The ventral surface of the centrum is markedly concave in lateral view (Fig. 2c,d). The centrum is slightly more than twice as long anteroposteriorly relative to the width of the posterior articular surface (Table 1). The centrum is amphicoelous. The central region of the anterior articular surface is flattened and surrounded laterally and ventrally by a concave rim (Fig. 2e), whereas the centre of the posterior articular surface is concave and bordered ventrally by a convex rim (Fig. 2f). The preserved portion of the anterior articular surface is elliptical in anterior view, wider mediolaterally than dorsoventrally tall (Fig. 2e). Only the ventralmost portion of the left parapophysis is present on the ventrolateral edge of the centrum anteriorly, and which also projects ventrolaterally (Fig. 2a,c,e). A region of exposed trabecular bone immediately dorsal to the preserved parapophysis indicates the likely size of its attachment to the centrum (Fig. 2c). An anteroposteriorly oriented lamina is present anterodorsally, extending from the anterior articular surface to approximately one third of the length of the centrum and overhanging the right lateral surface (Fig. 2a,b). The posterior edge of the lamina is broken, indicating that it likely continued further posteriorly. On the ventromedial surface of this lamina is the eroded remains of a smaller, vertically oriented lamina (Fig. 2d). The position of this smaller lamina would have been dorsal to the parapophysis, and its vertical and lateral continuation indicates that it would have contacted the diapophysis ventrally. Therefore, this lamina is interpreted as a paradiapophyseal lamina (ppdl; following nomenclature for vertebral laminae of Wilson^48^). Consequently, the portion of the larger lamina anterior to the ppdl is interpreted as the anterior centrodiapophyseal lamina (acdl), and the posterior portion is interpreted as the remains of the posterior centrodiapophyseal lamina (pcdl). The posterior articular surface is missing the dorsal portion due to erosion, similar to the anterior end, and is elliptical, having a greater mediolateral width than dorsoventral height (Fig. 2f). A portion of the floor of the neural canal is preserved across the anterior half of the dorsal surface of the centrum (Fig. 2a). Despite erosion to the dorsal surface of the centrum, the neural canal appears to have been mediolaterally wide, approximately half that of the centrum itself, and considerably wider than the neural arch pedicels as visible from their eroded bases (Fig. 2b). The ventral surface of the centrum is concave mediolaterally and delimited by well-defined, subparallel ventrolateral ridges that extend as laminae from the parapophyses along nearly the entire length of the centrum, becoming less distinct posteriorly (Fig. 2b). Two small (~3 mm long) lenticular foramina are present on the posterior half of the centrum (Fig. 2a). Whether these foramina are pneumatic in origin cannot be determined.

**Table 1:** Dimensions of LRF 3050.AR. Asterisks indicate incomplete measurements due to erosion or breakage.

Measurement	Value (mm)
Centrum, length along neural canal	75
Centrum, length along ventral surface	70
Centrum, minimum mediolateral width	29
Anterior articular surface, width	48
Anterior articular surface, height	22*
Posterior articular surface, width	40*
Posterior articular surface, height	18*

## Discussion

### Comparisons of LRF 3050.AR

Opisthocoelous vertebral centra characterise the cervical series of many neotheropods. The posterior surfaces are typically moderately to strongly concave and the anterior surface may be generally flattened^49,50^ or slightly convex as in ceratosaurians^51–54^ and basal tetanurans^55^, or form a well-defined projection as in abelisaurids^30,56^, megalosauroids^57–59^, allosauroids^49,60,61^, megaraptorids^9,62^ and alvarezsaurids^63–65^. In addition, opisthocoely continues into the anterior dorsal series in megalosauroids^57^, allosauroids^66,61^, megaraptorids^62^, and alvarezsaurids^63^. This differs from the condition in *Dilophosaurus wetherilli* and abelisauroids in which the anterior cervical centra are typically weakly opisthocoelous and transition along the series to amphicoelous in the most posterior cervicals and anterior dorsals^50–52,54,67,68^. All preserved mid-posterior cervical centra of *Elaphrosaurus* are amphicoelous^41^. Following these observations, the amphicoelous centrum and reduced inclination of the articular surfaces of LRF 3050.AR indicates a placement in the middle or posterior region of the neck. The distortion of the centrum, in particular the exaggerated offset of the articular surfaces resulting from taphonomic compression, precludes a more accurate placement of the centrum.

Among ceratosaurs, the dimensions of LRF 3050.AR are most similar to the anterior cervical series of the abelisaurid *Viavenator exxoni*. However, as noted above, the anterior cervical series in *Viavenator* and other abelisaurids consists of opisthocoelous centra, contrary to the amphicoelous condition in LRF 3050.AR. Unfortunately, direct comparisons of the centrum proportions of LRF 3050.AR are complicated by the strong taphonomic dorsoventral compression of the specimen. However, when the anterior half of the cervical centra are excluded, the dimensions of LRF 3050.AR are more similar to the moderately elongate proportions of noasaurids^41,68,69^ than the more robust and anteroposteriorly shortened centra in abelisaurids^51,52^ or strongly elongate centra in *Elaphrosaurus*^41^. The anterior and posterior articular surfaces are considerably wider mediolaterally than dorsoventrally tall (Table 1). This is similar to the proportions throughout the cervical series of *Masiakasaurus* *knopfleri* and *Elaphrosaurus*^41,67,68^, but may have been exaggerated by taphonomic distortion.

The preserved floor of the neural canal on the dorsal surface of LRF 3050.AR indicates that it was relatively wide mediolaterally relative to the width of the centrum and were likely wider than the thickness of the walls of the laterally bounding neural arch pedicels (Fig. 2). The neural canals in the cervicals of basal neotheropods and most ceratosaurs are narrower with respect to both the centrum and the neural arch pedicels^48,50,52,68^. In contrast, the neural canals of *Elaphrosaurus*^41^ and noasaurids^53,68,69^ are considerably wider relative to the centrum and wider than the thickness of the walls of the neural arch pedicels, as seen in LRF 3050.AR.

**Figure 3.**
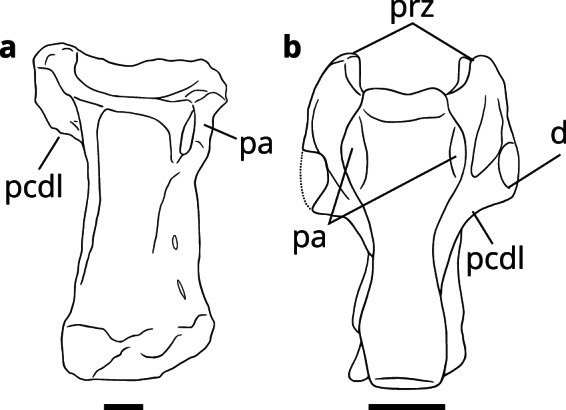
Comparisons of noasaurid cervical vertebrae. (**a**) LRF 3050.AR and (**b**) MACN-PV 622 (modified from Agnolín and Martinelli^53^) both in ventral view. Abbreviations as in Fig. 2 and prz, prezygapophyses. Scale bars equal 10 mm.

The distinct posterior centrodiapophyseal lamina (pcdl) of LRF 3050.AR is remarkably similar to those of noasaurids (Fig. 3). In MACN-PV (Museo Argentino de Ciencias Naturales “Bernardino Rivadavia”, Buenos Aires, Argentina) 622, a cervical vertebra initially described as an oviraptorosaur^70,71^ but which most likely pertains to *Noasaurus*^53^, the pcdl narrows abruptly from the anteriorly placed diapophyses and contacts the centrum at approximately the anteroposterior midpoint (Fig. 3). A similar pcdl also appears to have been present in GSI (Geological Survey of India, Kolkata, India) K20/614, a cervical vertebra ascribed to the Indian noasaurid *Laevisuchus indicus*^72^. The plesiomorphic condition of a posteriorly contacting pcdl is present in the middle cervicals of *Dilophosaurus*^50^, abelisauroids^30,34,54^ and also the recently described Brazilian noasaurid *Vespersaurus paranaensis*^69^. Despite the loss of the posterior portion of the posterior centrodiapophyseal lamina, a medial attachment of the pcdl is most likely to have been present in LRF 3050.AR. A medially positioned pcdl also characterises the middle to posterior cervical series of other ceratosaurs, including *Elaphrosaurus*^41^, *Majungasaurus crenatissimus*^52^ and *Carnotaurus sastrei*^51^.

Perhaps the most distinguishing feature of LRF 3050.AR is the mediolaterally concave ventral surface of the centrum delimited by pronounced ventrolateral ridges. In most ceratosaurs, the ventral surface of the cervical centra is flattened or slightly convex, forming a distinct edge at the contact with the lateral surfaces^51,68,73^. Ventrolateral ridges on cervical centra such as those present in LRF 3050.AR have been reported only in the basal ceratosaurian *Elaphrosaurus* and the noasaurid *Noasaurus*^41,53^. In *Elaphrosaurus*, the sharp lateroventrally directed ridges are present only at the posterior part of the centrum^41^, which differs from the condition in LRF 3050.AR in which they are continuous with the parapophysis and extend along almost the entire length of the centrum. Similar ventrolateral ridges have also been reported in MACN-PV 622^53^. Ventrolateral ridges have been described in therizinosaurs and unenlagiine dromaeosaurids^74–76^; however, they are developed only as comparatively weaker and rounded ridges that do not form the sharp edges that are seen in ceratosaurians. In addition, in unenlagiines the ventrolateral ridges transition into well-developed carotid processes at the anterior end of the centra^76,77^. This contrasts with the condition in LRF 3050.AR in which carotid processes are absent and the ridges remain sharply defined and contact the parapophyses at the anteroventral margins of the anterior articular surface.

### Status of NMV P221202

**Figure 4.**
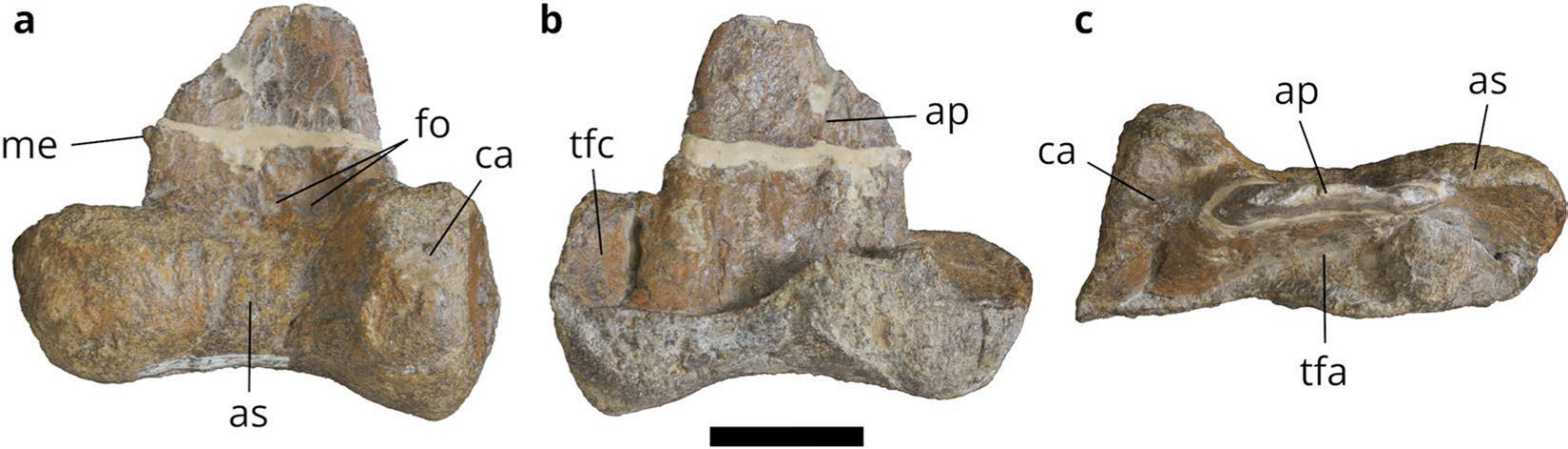
The astragalocalcaneum NMV P221202 in (**a**) anterior, (**b**) posterior, and (**c**) proximal views. Abbreviations: ap, ascending process of the astragalus; as, astragalar body; ca, calcaneum; fo, lateral anterior fossae; me, medial eminence; tfa, tibial facet of the astragalus; tfc, tibial facet of the calcaneum. Scale bar equals 20 mm.

A ceratosaurian astragalocalcaneum (NMV P221202) was discovered from the upper Barremian–lower Aptian San Remo Member of the upper Strzelecki group in Victoria^15^ (Fig. 4). NMV P221202 was compared to the only Australian theropod astragali known at the time, namely those of the megaraptorid *Australovenator wintonensis*^1^ and the Australian pygmy ‘*Allosaurus*’^78^, now considered to also pertain to Megaraptoridae^2,8^. The Victorian astragalocalcaneum, NMV P221202, was found to differ from the two Australian megaraptorid astragali, most notably in the co-ossification of the astragalus and calcaneum, the absence of a horizontal vascular groove on the anterior surface of the astragalar body, and the lack of a crescentic groove on the posterior surface of the ascending process^15^. NMV P221202 was referred to Ceratosauria in a phylogenetic analysis, but possible ingroup relationships were not considered with confidence despite similarities with the astragalus of the Madagascan noasaurid *Masiakasaurus*^15^.

Subsequently, the assignment of NMV P221202 to Ceratosauria was questioned^8^ on the basis of five observations: the presence of a distinct eminence on the medial surface of the ascending process and paired oval fossae at the base of the ascending process of the astragalus anteriorly (Fig. 4a), both of which are present in alvarezsaurids^79^; a vertical groove on the posterior surface of the ascending process and a lateral constriction of the tibial facet caused by a thickening of the ascending process laterally (Fig. 4c), both of which are present in megaraptorids; and a prominent posterodorsal notch on the calcaneum for articulation of the tibia (Fig. 4b), which they considered to be a tetanuran synapomorphy based on the results of a phylogenetic analysis of tetanurans^80^. Based on these observations, it was concluded that NMV P221202 could only be considered an indeterminate averostran^8^. The debate surrounding the affinities of NMV P221202 was commented on briefly in a review of the Victorian Cretaceous polar biota^81^, with no preference stated for either of the two hypotheses.

However, a detailed consideration of these arguments as presented raises a number of problems. Firstly, as previously noted^8^, the ascending process of the astragalus in alvarezsaurids differs markedly from the condition present in NMV P221202. As is typical for coelurosaurs, the base of the ascending process in alvarezsaurids occupies almost the entire width of the astragalus^63,79^. Furthermore, in alvarezsaurids with the exception of *Patagonykus puertai*, the medial surface of the ascending process is excavated by a deep notch, leaving only a low medial portion of the ascending process and a taller narrow lateral portion^63,65,82–84^. However, in NMV P221202 the ascending process is parallel-sided at the base, was likely subrectangular in its original form, and its base spans only the lateral two-thirds of the astragalus. In addition, contrary to previous remarks^8^, no medial eminence of the ascending process that resembles that of NMV P221202 is present in either *Patagonykus* or *Mononykus olecranus*. In the former taxon, the medial edge of the ascending process is smoothly sinusoidal in anterior view with no noticeable eminences^79^, whereas the medial edges of the medially-notched ascending processes of *Mononykus* and other alvarezsaurids are straight or slightly concave, with no noticeable eminences^63,65^. Secondly, as noted in the original description of NMV P221202^15^, and contrary to previous observations^8^, there is no groove on the posterior surface of the ascending process similar to those that have been reported in megaraptorids. The lateral edge of the posterior surface of the base of the ascending process in NMV P221202 is slightly elevated with respect to the area immediately lateral to an abraded area of periosteum that may have given the appearance of a grooved surface. However, this is markedly different from the well-defined crescentic groove present on the posterior surface of the ascending process in megaraptorid astragali^1,14,78^. Thirdly, the lateral side of the tibial facet of the astragalus in the abelisaurid *Majungasaurus* is also constricted relative to the medial side^85^, indicating that this feature is not restricted to megaraptorids as previously asserted^8^ and that abelisauroid affinities cannot be dismissed. Finally, tibial facets on the calcaneum have been observed in *Dilophosaurus*, *Majungasaurus*, *Elaphrosaurus*, *Ceratosaurus* and *Masiakasaurus*^41,67,85,86^, indicating that this feature is diagnostic of Averostra, a more inclusive group than stated previously^8^.

### Phylogenetic analysis

**Figure 5.**
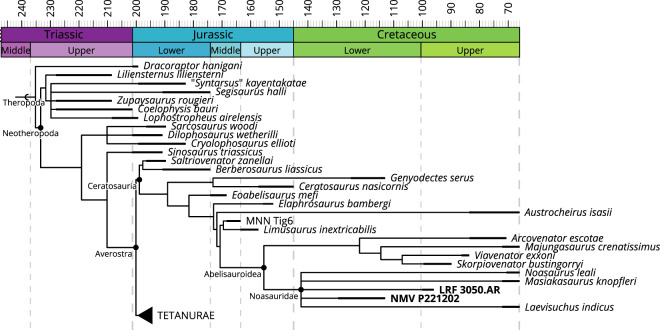
Strict consensus tree of the modified ceratosaurian phylogenetic matrix of Dal Sasso *et al*.^39^ highlighting the phylogenetic positions of the Australian noasaurid specimens LRF 3050.AR and NMV P221202.

The phylogenetic analysis including LRF 3050.AR and NMV P221202 (see Methods and Materials for details) returned 217 most parsimonious trees of 4293 steps (CI: 0.306, RI: 0.512). The strict consensus tree resolves both Australian specimens within Noasauridae (Fig. 5). The synapomorphies diagnosing Noasauridae include a spur on the medial surface of the ascending process of the astragalus (858:1), mediolaterally thin cervical epipophyses (1272:1), cervical postzygapophyses swept back posteriorly and surpassing the posterior end of the vertebral centra (1083:1), smooth medial surfaces of the anteromedial process of the maxilla (915:0), anteroposteriorly shortened palatal shelves of the maxilla (1310:1), paradental plates of the maxilla low and partially obscured by lamina of maxilla (972:1) and shaft of metatarsal II mediolaterally compressed (1208:1). The presence of ventrolateral ridges contacting the parapophyses on the cervical vertebrae (210:1) may represent an additional synapomorphy of Noasauridae. However, the distribution of this character is presently uncertain and so far has only been reported in MACN-PV 622 (cf. *Noasaurus*), in addition to LRF 3050.AR. The noasaurid with the most complete cervical series, *Masiakasaurus*, has flattened ventral surfaces of the centra with no ventrolateral ridges^41^. When *Masiakasaurus* is coded as such for the aforementioned character, the presence of ventrolateral ridges does not optimise as a synapomorphy of Noasauridae. However, this may be an artifact of the long-standing lack of resolution among noasaurids due to their poor fossil record, and it remains plausible that ventrolateral ridges may represent a synapomorphy of a subclade within Noasauridae. However, more data is needed to thoroughly test this hypothesis.

The presence of a medial eminence on the ascending process is a synapomorphy that pertains directly to NMV P221202. Among theropods, this feature is shared only with *Masiakasaurus*^68^ and represents the strongest evidence in favour of noasaurid affinities for NMV P221202. Unfortunately, the lack of preserved ascending processes in the astragali of other noasaurid taxa precludes detailed comparisons.

If the results presented here are correct, then NMV P221202 and LRF 3050.AR represent novel reports of noasaurids from the late Barremian–early Aptian of Victoria and Cenomanian of New South Wales respectively. Under the taxonomic framework presented here, Noasauridae consists of at least six named taxa: *Laevisuchus*, *Noasaurus* and *Masiakasaurus* from the Maastrichtian of India, Argentina, and Madagascar respectively^44,67,87^; *Velocisaurus*, from the Santonian of Argentina^88^; *Vespersaurus* from the Aptian–Campanian of Brazil^69^ and *Afromimus tenerensis* from the Aptian–Albian of Niger, initially described as an ornithomimid^89^ but recently reappraised as a probable noasaurid^90^. *Genusaurus sisteronis*, from the Albian of France, has previously been considered as a noasaurid^22^, but subsequent analyses, including the one presented here, preferred a position within Abelisauridae. *Ligabueino andesi*, from the Barremian–early Aptian of Argentina^91^, was also originally described as a noasaurid, but phylogenetic studies failed to identify any noasaurid synapomorphies in this taxon^22,68^. NMV P221202, which is identified by phylogenetic analysis as a noasaurid, therefore represents the oldest known representative of the clade in the world to date (Fig. 5). However, if the broader taxonomic scope of Noasauridae (i.e., inclusive of elaphrosaurines; see Taxonomic Framework) is favoured instead, then NMV P221202 would instead represent the oldest known noasaurine, with the oldest noasaurids represented by the Middle–Late Jurassic aged elaphrosaurines^33,41,92^. Regardless of their phylogenetic position, the newly described Australian noasaurids expands the known palaeogeographic range of the clade outside of South America, Madagascar and India. Presently, the poor fossil record of Noasauridae, and the corresponding lack of resolution among the known noasaurid taxa, precludes the formation of any novel palaeobiogeographic hypotheses including the newly discovered Australian record of noasaurid theropods. Future discoveries may reveal more detail about the evolution and palaeobiogeographic distribution of this enigmatic clade.

## Methods and Materials

LRF 3050.AR and NMV P221202 were inserted into a recently published ceratosaurian phylogenetic matrix^39^ (see Supplementary Dataset S1) and analysed with equal weights parsimony in TNT 1.5^93^. A driven search strategy was implemented to calculate optimal trees, with each search using 100 replicates of random sectorial searches, each with 30 rounds of drifting, 5 rounds of tree fusing and 50 ratcheting cycles. The analysis was halted after two such successive searches returned shortest trees of the same length.

### Supplementary information

Supplementary information

## Data Availability

All data generated or analysed during this study are included in this published article (and its Supplementary Information).
