# Supplementary material for: Noasaurids are a component of the Australian ‘mid’-Cretaceous theropod fauna
Source: Sci Rep. 2020 Jan 29;10:1428. doi: 10.1038/s41598-020-57667-7 (PMC6989633; doi:10.1038/s41598-020-57667-7)
Supplement: Supplementary file 1 — Supplementary information [file 41598_2020_57667_MOESM1_ESM.zip › Supplementary_Information.pdf]

Supplementary information for “Noasaurids are a component of the Australian ‘mid’-Cretaceous theropod fauna”

Sienna A. Birch[1], Elizabeth T. Smith[2], and Phil R. Bell[1]

[1]School of Environmental and Rural Science, Universiy of New England, Armidale, NSW, Australia

[2]Australian Opal Centre, Lightning Ridge, NSW, Australia

Contents

1 List of taxa compared **2**

2 Matrix modifications **5**

3 Complete tree **6**

Supplementary References **7**

1 List of taxa compared

Supplementary Table 1: A list of the theropod taxa and specimens compared with LRF 3050.AR and NMV P221202 in the assessment of their affinities, together with their age ranges and sources.

Taxon	Specimen(s)	Age range	Source
Acrocanthosaurus atokensis	SMU 74646	Aptian	Harris (1998)
Aerosteon riocoloradensis	MCNA-PV-3137	Early Campanian–Middle Campanian	Sereno et al. (2008)
Allosaurus fragilis	USNM 8367	Kimmeridgian–Tithonian	Gilmore (1920)
Australovenator wintonensis	AODF 604	Late Cenomanian	Hocknull et al. (2009)
Baryonyx walkeri	BMNH R9951	Early Barremian	Charig and Milner (1997)
Carnotaurus sastrei	MACN-CH-894	Late Campanian–Early Maastrichtian	Bonaparte et al. (1990); Méndez (2014)
Ceratosaurus nasicornis	USNM 4735	Kimmeridgian–Tithonian	Gilmore (1920)
Dahalokely tokana	UA 9855	Turonian	Farke and Sertich (2013)
Dilophosaurus wetherilli	UCMP 37302, TMM 43646	Sinemurian–Pliensbachian	Welles (1984); Tykoski (2005)
Ekrixinatosaurus novasi	MUCPv-294	Early Cenomanian	Calvo et al. (2004)
Elaphrosaurus bambergi	MB R 4960	Late Kimmeridgian	Rauhut and Carrano (2016)
Eoabelisaurus mefi	MPEF PV 3990	Aalenian–Early Bajocian	Pol and Rauhut (2012)

Taxon	Specimen(s)	Age range	Source
Eustreptospondylus oxoniensis	OUMNH J.13558	Late Callovian	Sadleir et al. (2008)
Ilokelesia aguadagrandensis	PVPH-35	Late Cenomanian–Turonian	Coria and Salgado (2000)
Laevisuchus indicus	GSI K20/613–614	Maastrichtian	von Huene and Matley (1933); Novas et al. (2004)
Linhenykus monodactylus	IVPP V17608	Campanian	Xu et al. (2013)
MNN Tig6	MNN Tig6	Bathonian–Callovian	Sereno et al. (2004)
Majungasaurus crenatissimus	UA 8678	Maastrichtian	O'Connor (2007)
Masiakasaurus knopfleri	FMNH PR 2140–2141, 2481; UA 9106	Maastrichtian	Carrano et al. (2002); Carrano et al. (2011)
Megaraptor namunhuaiquii	MUCPv 595	Late Turonian–Early Coniacian	Porfiri et al. (2014)
Monolophosaurus jiangi	IVPP 84019	Callovian	Zhao et al. (2010)
Mononykus olecranus	IGM 107/6	Maastrichtian	Perle et al. (1994)
MACN PV-622 (*Noasaurus leali*)	MACN PV-622	Early Maastrichtian	Agnolin and Martinelli (2007)
Patagonykus puertai	PVPH-37	Late Turonian–Early Coniacian	Novas (1997)
Shuvuuia deserti	IGM 100/975	Campanian	Chiappe et al. (2002)
Sinraptor dongi	IVPP 10600	Oxfordian	Currie and Zhao (1993)

Taxon	Specimen(s)	Age range	Source
Torvosaurus tanneri	BYUVP 4860, BYUVP 2004a—d	Late Kimmeridgian–Early Tithonian	Britt (1991)
Velocisaurus unicus	MUCPv-41	Santonian	Bonaparte (1991)
Vespersaurus paranaensis	MPCO.V 0017, 0034, 0035, 0048	Aptian–Campanian	Langer et al. (2019)
Viavenator exxoni	MAU-Pv-LI-530	Santonian	Filippi et al. (2018)

2 Matrix modifications

The following modifications and additions were made to the phylogenetic matrix of Dal Sasso et al. (2018) for evaluating the affinities of LRF 3050.AR and NMV P221202:

1. *Laevisuchus* was added following the descriptions in Novas et al. (2004);
2. The character statement for character 210 was changed to "Cervical vertebrae, post-axial centra, ventral sulcus delimited by ventrolaterally directed ridges **that contact parapophyses**". This change in definition reflects the character state as present in MACN PV 622 (cf. *Noasaurus*) and LRF 3050.AR. The ceratosaurians *Elaphrosaurus* and MNN Tig6 and the basal sauropodomorph *Panphagia* were scored as present for this character. This character was regarded as absent in MNN Tig6 and ventrolateral ridges in *Elaphrosaurus* are present only on the posteriormost extent of the centra (Rauhut and Carrano 2016). The purported ventrolateral ridges in the cervical vertebrae of *Panphagia* diverge from a single ventral keel and extending anteriorly without contacting the parapophyses (Martinez and Alcober 2009). Therefore, the following coding changes here made:
   - *Elaphrosaurus*: 1 $\rightarrow$ 0 (after Rauhut and Carrano 2016)
   - MNN Tig6: 1 $\rightarrow$ 0 (after Rauhut and Carrano 2016)
   - *Noasaurus*: ? $\rightarrow$ 1 (after Agnolin and Martinelli 2007)
   - *Panphagia*: 1 $\rightarrow$ 0 (after Martinez and Alcober 2009)
3. *Masiakasaurus* was scored as having a fibular articular facet on the ascending process of the astragalus (530:1; following Carrano et al. 2002, 2011).
4. *Majungasaurus* and *Masiakasaurus*were scored as having 'V'- and 'U'-shaped cervical infraprezygapophyseal space in dorsal view respectively (1212:0 and 1212:1; following O'Connor 2007; Carrano et al. 2002, 2011).

3 Complete tree

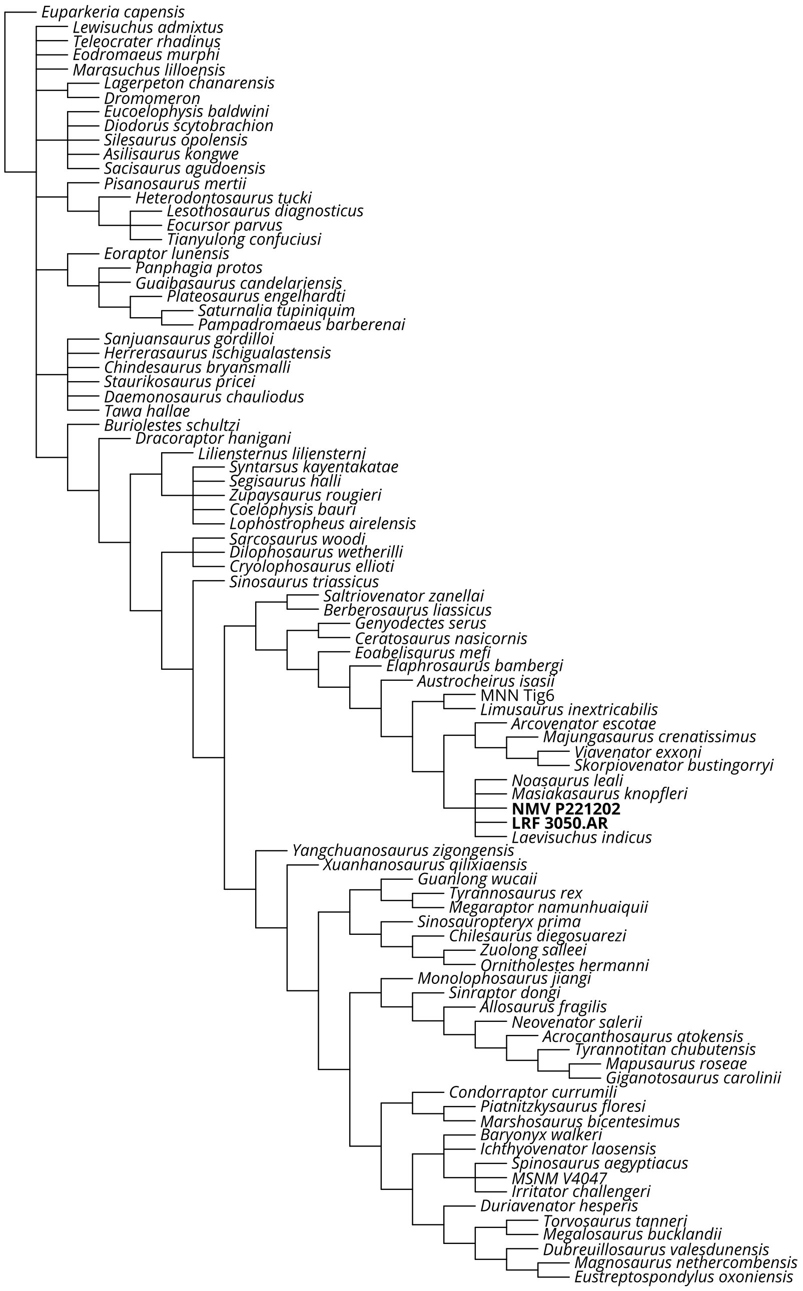

Supplementary Figure 1: Complete phylogenetic tree resulting from analysis of the modified version of a ceratosaurian phylogenetic matrix of Dal Sasso et al. (2018) including the Australian noasaurid specimens LRF 3050.AR and NMV P221202.

Supplementary References

Agnolin, F. L., & Martinelli, A. G. (2007). Did oviraptorosaurs (Dinosauria; Theropoda) inhabit Argentina? *Cretaceous Research*, *28*(5), 785–790. https://doi.org/10.1016/j.cretres.2006.10.006

Bonaparte, J. F. (1991). Los vertebrados fósiles de la Formación Rio Colorado, de la ciudad de Neuquén y cercanias, Cretácico superior, Argentina. *Revista del Museo argentino de ciencias naturales "Bernardino Rivadavia" e Instituto nacional de investigacion de las ciencias naturales, Paleontologia*, *4*(3).

Bonaparte, J. F., Novas, F., & Coria, R. (1990). *Carnotaurus sastrei* Bonaparte, the horned, lightly built carnosaur from the Middle Cretaceous of Patagonia. *Contrib. in sci./Natural history nuseum of Los Angeles county*, *416*, 1–41.

Britt, B. B. (1991). Theropods of Dry Mesa Quarry (Morrison Formation, Late Jurassic), Colorado, with emphasis on the osteology of *Torvosaurus tanneri*. *Brigham Young University Geology Studies*, *37*, 1–72.

Calvo, J. O., Rubilar-Rogers, D., & Moreno, K. (2004). A new Abelisauridae (Dinosauria: Theropoda) from northwest Patagonia. *Ameghiniana*, *41*(4), 555–563.

Carrano, M. T., Loewen, M. A., & Sertich, J. J. (2011). New materials of *Masiakasaurus knopfleri* Sampson, Carrano, and Forster, 2001, and implications for the morphology of the Noasauridae (Theropoda: Ceratosauria). *Smithsonian Contributions to Paleontology*, *95*, 1–53.

Carrano, M. T., Sampson, S. D., & Forster, C. A. (2002). The osteology of *Masiakasaurus knopfleri*, a small abelisauroid (Dinosauria: Theropoda) from the Late Cretaceous of Madagascar. *Journal of Vertebrate Paleontology*, *22*(3), 510–534.

Charig, A. J., & Milner, A. C. (1997). *Baryonyx walkeri*, a fish-eating dinosaur from the Wealden of Surrey. *Bulletin of the Natural History Museum London*, *53*(1), 11–70.

Chiappe, L. M., Norell, M. A., & Clark, J. M. (2002). The Cretaceous short-armed Alvarezsauridae: *Mononykus* and its kin. In L. M. Chiappe & L. M. Witmer (Eds.), *Mesozoic Birds: Above the Heads of Dinosaurs* (pp. 87–120). University of California Press.

Coria, R. A., & Salgado, L. (2000). A basal Abelisauria Novas, 1992 (Theropoda-Ceratosauria) from the Cretaceous of Patagonia, Argentina. *Gaia*, *15*, 89–102.

Currie, P. J., & Zhao, X.-J. (1993). A new carnosaur (Dinosauria, Theropoda) from the Jurassic of Xinjiang, People's Republic of China. *Canadian Journal of Earth Sciences*, *30*(10), 2037–2081. https://doi.org/10.1139/e93-179

Dal Sasso, C., Maganuco, S., & Cau, A. (2018). The oldest ceratosaurian (Dinosauria: Theropoda), from the Lower Jurassic of Italy, sheds light on the evolution of the three-fingered hand of birds. *PeerJ*, *6*, e5976. https://doi.org/10.7717/peerj.5976

Farke, A. A., & Sertich, J. J. W. (2013). An abelisauroid theropod dinosaur from the Turonian of Madagascar. *PLOS ONE*, *8*(4), e62047. https://doi.org/10.1371/journal.pone.0062047

Filippi, L. S., Méndez, A. H., Gianechini, F. A., Juárez Valieri, R. D., & Garrido, A. C. (2018). Osteology of *Viavenator exxoni* (Abelisauridae; Furileusauria) from the Bajo de la Carpa Formation, NW Patagonia, Argentina. *Cretaceous Research*, *83*, 95–119. https://doi.org/10.1016/j.cretres.2017.07.019

Gilmore, C. W. (1920). Osteology of the carnivorous Dinosauria in the United States National Museum, with special reference to the genera *Antrodemus* (*Allosaurus*) and *Ceratosaurus*. *Bulletin of the United States National Museum*, *110*, 1–159.

Harris, J. D. (1998). A reanalysis of *Acrocanthosaurus atokensis*, its phylogenetic status, and paleobiogeographic implications, based on a new specimen from Texas. *New Mexico Museum of Natural History and Science Bulletin*, *13*, 1–75.

Hocknull, S. A., White, M. A., Tischler, T. R., Cook, A. G., Calleja, N. D., Sloan, T., & Elliott, D. A. (2009). New mid-Cretaceous (latest Albian) dinosaurs from Winton, Queensland, Australia. *PLoS ONE*, *4*(7), e6190. https://doi.org/10.1371/journal.pone.0006190

Langer, M. C., Martins, N. d. O., Manzig, P. C., Ferreira, G. d. S., Marsola, J. C. d. A., Fortes, E., Lima, R., Sant'ana, L. C. F., Vidal, L. d. S., Lorençato, R. H. d. S., & Ezcurra, M. D. (2019). A new desert-dwelling dinosaur (Theropoda, Noasaurinae) from the Cretaceous of south Brazil. *Scientific Reports*, *9*(1), 9379. https://doi.org/10.1038/s41598-019-45306-9

Martinez, R. N., & Alcober, O. A. (2009). A Basal Sauropodomorph (Dinosauria: Saurischia) from the Ischigualasto Formation (Triassic, Carnian) and the Early Evolution of Sauropodomorpha (P. Sereno, Ed.). *PLoS ONE*, *4*(2), e4397. https://doi.org/10.1371/journal.pone.0004397

Méndez, A. (2014). The cervical vertebrae of the Late Cretaceous abelisaurid dinosaur *Carnotaurus sastrei*. *Acta Palaeontologica Polonica*, *59*(3), 569–579. https://doi.org/10.4202/app.2012.0095

Novas, F., Agnolín, F. L., & Bandyopadhyay, S. (2004). Cretaceous theropods from India: A review of specimens described by Huene and Matley (1933). *Revista del Museo Argentino de Ciencias Naturales nueva serie*, *6*(1), 67–103.

Novas, F. E. (1997). Anatomy of *Patagonykus puertai* (Theropoda, Avialae, Alvarezsauridae), from the Late Cretaceous of Patagonia. *Journal of Vertebrate Paleontology*, *17*(1), 137–166. https://doi.org/10.1080/02724634.1997.10010959

O'Connor, P. M. (2007). The postcranial axial skeleton of *Majungasaurus crenatissimus* (Theropoda: Abelisauridae) from the Late Cretaceous of Madagascar. *Journal of Vertebrate Paleontology*, *27*(S2), 127–163.

Perle, A., Chiappe, L. M., Barsbold, R., Clark, J. M., & Norell, M. A. (1994). Skeletal morphology of *Mononykus olecranus* (Theropoda: Avialae) from the Late Cretaceous of Mongolia. *American Museum Novitates*, *3105*, 1–29.

Pol, D., & Rauhut, O. W. M. (2012). A Middle Jurassic abelisaurid from Patagonia and the early diversification of theropod dinosaurs. *Proceedings of the Royal Society B: Biological Sciences*, *279*(1741), 3170–3175. https://doi.org/10.1098/rspb.2012.0660

Porfiri, J. D., Novas, F. E., Calvo, J. O., Agnolín, F. L., Ezcurra, M. D., & Cerda, I. A. (2014). Juvenile specimen of *Megaraptor* (Dinosauria, Theropoda) sheds light about tyrannosauroid radiation. *Cretaceous Research*, *51*, 35–55. https://doi.org/10.1016/j.cretres.2014.04.007

Rauhut, O. W. M., & Carrano, M. T. (2016). The theropod dinosaur *Elaphrosaurus bambergi* Janensch, 1920, from the Late Jurassic of Tendaguru, Tanzania. *Zoological Journal of the Linnean Society*, *178*(3), 1–65. https://doi.org/10.1111/zoj.12425

Sadleir, R. W., Barrett, P. M., & Powell, H. P. (2008). The anatomy and systematics of *Eustreptospondylus oxoniensis*. *Monograph of the Palaeontographical Society, London*, *160*, 1–82.

Sereno, P. C., Wilson, J. A., & Conrad, J. L. (2004). New dinosaurs link southern landmasses in the Mid-Cretaceous. *Proceedings of the Royal Society B: Biological Sciences*, *271*(1546), 1325–1330. https://doi.org/10.1098/rspb.2004.2692

Sereno, P. C., Martinez, R. N., Wilson, J. A., Varricchio, D. J., Alcober, O. A., & Larsson, H. C. E. (2008). Evidence for avian intrathoracic air sacs in a new predatory dinosaur from Argentina. *PLoS ONE*, *3*(9), e3303. https://doi.org/10.1371/journal.pone.0003303

Tykoski, R. S. (2005). *Anatomy, ontogeny, and phylogeny of coelophysoid theropods* [Doctoral dissertation].

von Huene, F., & Matley, C. A. (1933). The Cretaceous Saurischia and Ornithischia of the central provinces of India. *Memoirs of the Geological Survey of India*, *21*(1), 1–74.

Welles, S. P. (1984). *Dilophosaurus wetherilli* (Dinosauria, Theropoda) osteology and comparisons. *Palaeontographica Abteilung A*, *185*(4-6), 85–180.

Xu, X., Upchurch, P., Ma, Q., Pittman, M., Choiniere, J. N., Sullivan, C., Hone, D. W. E., Tan, Q., Tan, L., Xiao, D., & Han, F. (2013). Osteology of the alvarezsauroid *Linhenykus monodactylus* from the Upper Cretaceous Wulansuhai Formation of Inner Mongolia, China, and comments on alvarezsauroid biogeography. *Acta Palaeontologica Polonica*, *58*(1), 25–46. https://doi.org/10.4202/app.2011.0083

Zhao, X., Benson, R. B. J., Brusatte, S. L., & Currie, P. J. (2010). The postcranial skeleton of *Monolophosaurus jiangi* (Dinosauria: Theropoda) from the Middle Jurassic of Xinjiang, China, and a review of Middle Jurassic Chinese theropods. *Geological Magazine*, *147*(1), 13–27. https://doi.org/10.1017/S0016756809990240
